# Parental stress, coping strategies, and maternal quality of life in urban Tanzania: A mixed-methods study

**DOI:** 10.1371/journal.pone.0358956

**Published:** 2026-09-25

**Authors:** Hye-Kyung Oh, Boram Lee

**Affiliations:** 1 College of Nursing, Daegu University, Daegu, Republic of Korea; 2 Department of Child and Family Welfare, Daegu University, Gyeongsan-si, Republic of Korea; The Open University of Israel, ISRAEL

## Abstract

**Purpose:**

Maternal quality of life (QoL) is influenced by psychosocial and socioeconomic challenges, particularly in low-resource settings. However, little is known about how parental stress and coping strategies affect maternal well-being in Tanzania. This study examined the relationships among parental stress, coping strategies, and QoL among mothers living in Kigamboni, Tanzania, using a convergent mixed-methods approach.

**Methods:**

A convergent mixed-methods design was employed, combining quantitative survey data from 186 mothers with qualitative interviews involving nine stakeholders, including parents, educators, and local government officials. The survey assessed parental stress, coping strategies, and QoL. Quantitative data were analyzed using descriptive statistics, independent t-tests, Pearson’s correlation coefficients, and multiple linear regression analyses. Qualitative data were analyzed using inductive thematic analysis.

**Results:**

Problem-focused and emotion-focused coping strategies were associated with better QoL. In contrast, avoidant coping was negatively associated with maternal well-being. Several sociodemographic factors, including having a child with a disability, lower paternal education, lower household wealth, and single parenthood, were associated with higher levels of parental stress and maladaptive coping. Although parental stress showed a negative bivariate association with quality of life, it did not remain a significant independent predictor in the multivariable model. Qualitative findings identified three major themes: (1) parenting stress as a daily struggle, (2) adaptive and strained coping strategies, and (3) structural and cultural barriers to maternal well-being. Participants described chronic financial hardship, emotional exhaustion, social stigma toward disability, and limited formal and informal support systems as important influences on maternal QoL.

**Conclusion:**

Maternal quality of life in Tanzania is shaped by the interplay between psychosocial factors and broader social and structural conditions. The integrated findings suggest that improving maternal well-being requires multilevel interventions that strengthen adaptive coping resources while addressing parenting-related stress and broader contextual barriers.

## Introduction

Tanzania is a country in the African region and is relatively politically and socially stable, with high development potential [1]. Although Tanzania’s poverty rate is declining, strong population growth is causing the total number of people in poverty to rise [2]. Poverty has a significant impact on reducing maternal responsiveness and increasing the parental stress of mothers [3]. Specifically, women remain a socially and economically disadvantaged group compared with men, experiencing lower economic status, higher unemployment, and fewer educational opportunities in Tanzania [1]. Mothers are the primary caregivers of children [4], and these socioeconomic disadvantages may place them at increased risk of psychological distress and reduced quality of life while raising their children [1–3].

Parental stress is defined as the difficulties and challenges of parenting while caring for their children [5]. Previous studies have shown that economic factors such as unemployment and housing conditions are associated with higher parental stress [6], and poverty has consistently been identified as a major contributor to parenting-related stress [7]. Vulnerability factors, including limited income and educational opportunities, further increase parental stress [6,8]. Parental stress has also been identified as one of the strongest predictors of maternal quality of life and parent-child relationships [9–13]. Therefore, understanding parental stress is essential for improving maternal well-being, particularly in low-resource settings such as Tanzania. According to the Transactional Model of Stress and Coping, the impact of stressful experiences depends not only on the stressors themselves but also on how individuals appraise and cope with them [14,15]. Accordingly, coping strategies may represent an important psychosocial factor associated with maternal quality of life among mothers experiencing parenting-related stress.

To reduce parenting-related stress, mothers adopt various coping strategies, which refer to the cognitive and behavioral efforts used to manage stressful, difficult, or challenging situations [16]. These strategies are generally categorized as problem-focused, emotion-focused, and avoidant coping [17]. The effectiveness of these coping strategies varies considerably, resulting in different levels of psychological adjustment and quality of life. Previous studies have shown that avoidant coping is associated with poorer mental health outcomes [18–20], whereas problem-focused coping is associated with lower distress and better quality of life among mothers [21]. Together, parental stress and coping strategies represent important psychosocial factors associated with maternal quality of life.

Quality of life is defined as an individual’s perception of their position in life in the context of the culture and value systems in which they live, and their goals, expectations, standards, and concerns [22]. Quality of life is a key indicator that determines overall functioning and well-being in mothers, and it highlights important directions for health interventions [23]. Moreover, it goes beyond simple health outcomes and serves as a key tool for evaluating the effectiveness of health interventions and informing policy decisions [24]. Despite growing evidence regarding parental stress, coping strategies, and maternal quality of life, most existing studies have been conducted in high-income countries, with relatively limited evidence available from low-resource settings such as Tanzania. Furthermore, previous research has primarily focused on quantitative associations among these variables, providing a limited understanding of how mothers experience parenting-related stress and coping within their everyday social and economic contexts.

Furthermore, little is known about how these quantitative relationships are experienced and interpreted by mothers within the social and cultural context of Tanzania, limiting the development of culturally appropriate maternal mental health interventions. Because maternal quality of life is influenced not only by measurable psychosocial factors but also by contextual and lived experiences [3,9,12], quantitative findings alone may not provide a comprehensive understanding of mothers’ well-being [25,27]. Integrating quantitative and qualitative evidence can therefore offer a more complete understanding by identifying statistical relationships while explaining the contextual experiences underlying them [25–27].

Accordingly, this study employed a mixed-methods approach to examine the relationships among parental stress, coping strategies, and maternal quality of life among mothers living in Kigamboni, Tanzania. Specifically, the quantitative component identified psychosocial factors associated with maternal quality of life, whereas the qualitative component explored mothers’ and stakeholders’ perspectives to explain and contextualize these quantitative findings and to inform the development of community-based maternal mental health interventions in Tanzania.

## Materials and methods

### Study design

This study employed a convergent mixed-methods design to obtain a comprehensive understanding of the factors associated with maternal quality of life in Tanzania [25–27]. Quantitative and qualitative data were collected during the same period, analyzed separately, and subsequently integrated during interpretation. The quantitative component examined the associations among parental stress, coping strategies, and maternal quality of life, whereas the qualitative component explored contextual experiences and perspectives that could explain and enrich the quantitative findings. The study assigned equal priority to the quantitative and qualitative components, and findings from both strands were integrated at the interpretation stage through comparison and triangulation to identify areas of convergence and complementarity. The integration of both components enabled a more comprehensive understanding of maternal well-being in the Tanzanian context.

### Participants

Participants were recruited from a health center and a primary school located in the Kigamboni area using a convenience sampling approach. Mothers who met the inclusion criteria and were present at the recruitment sites during the data collection period were invited to participate voluntarily. The inclusion criteria were as follows: (a) being a mother aged 18 years or older, (b) currently raising at least one child, (c) residing in the Kigamboni district, and (d) being able to communicate in Swahili. Mothers who were unable to provide informed consent or who did not complete the questionnaire were excluded from the analysis. Data were collected at different times of the day to capture mothers with varying daily routines. Although efforts were made to include a diverse group of mothers, the sample may not be fully representative of all mothers in Kigamboni or other regions of Tanzania. Therefore, the findings should be interpreted with caution in terms of generalizability beyond the study setting.

The survey was administered from April 22–30, 2023. The questionnaire was written in Swahili. For the quantitative research, the required sample size was calculated as 146 using G*Power 3.1.9.2, based on a medium effect size (0.15), a significance level of 0.05, and a statistical power of 0.95. To account for potential attrition, a total of 200 participants were enrolled. After data collection, incomplete, invalid, or non-target responses were excluded during data cleaning, resulting in a final analytic sample of 186 participants.

For the qualitative component, one-on-one semi-structured interviews were conducted with stakeholders who were expected to provide contextual information regarding parenting experiences, cultural beliefs, and formal and informal support systems relevant to maternal quality of life. Purposive sampling was used to recruit participants with diverse roles and experiences that could aid in interpreting the quantitative findings. A total of nine stakeholders, one local district commissioner, one officer in the educational department of local government, two primary school principals, three primary school teachers, and two parents were interviewed during the same period as the quantitative survey. The inclusion of multiple stakeholder groups was intended to provide complementary perspectives on the social and community contexts surrounding motherhood and parenting in Tanzania, thereby facilitating a more comprehensive interpretation of the quantitative findings. All participants, with the exception of one local district commissioner, were married and actively raising children.

### Research Ethics

This study was approved by the Institutional Review Board of Daegu University (Approval No. 1040621–202303-HR-033). Before data collection, written authorization for the study was documented through a Letter of Intent signed by the District Commissioner following a meeting at which the research objectives, procedures, institutional ethical approval, and participant consent process were explained. Through the District Commissioner’s Office, permission to conduct the study was also obtained from the director of Kigamboni Health Centre. Prior to participation, all participants received detailed information regarding the study objectives, procedures, confidentiality, and voluntary participation. Participants were informed that they could withdraw from the study at any time without penalty. Written informed consent was obtained from all participants before data collection. Data collection was conducted with the assistance of trained Tanzanian research assistants who were fluent in Swahili, facilitated communication with participants, and helped ensure culturally appropriate implementation of the study. The study was conducted in accordance with local customs and institutional procedures while adhering to the ethical standards approved by the Institutional Review Board of Daegu University.

### Measurements

The eight items concerning participants’ personal characteristics were constructed by researchers based on previous studies, and focused on age, participants’ and father’s education level, marital status, the number of children in the household, whether the child has a disability, the number of income-generating activities in the family, and the family’s wealth index. All instruments, including the participants’ personal characteristics questionnaire and measures of the main study variables, were originally developed in English and translated into Swahili before the survey. The translation was performed by an independent expert with a doctoral degree who was fluent in both English and Swahili. The translated version was then back-translated into English by another bilingual expert who had not been involved in the initial translation. To ensure linguistic accuracy, conceptual equivalence, and cultural appropriateness, the original and back-translated versions were compared, and any discrepancies were resolved through discussion. The final Swahili version was subsequently reviewed by local health professionals familiar with the Tanzanian context. The complete survey questionnaires in both Swahili and English are provided as Supporting Information (S1 File). Additional information regarding the ethical, cultural, and scientific considerations specific to inclusivity in global research is included in the Supporting Information (S2 File).

### Quality of life

The level of quality of life was measured using the WHOQOL scale [22], which consists of 4 subscales: physical health, psychological health, social relationships, and environment. Each item was rated on a 5-point Likert scale. Higher score indicates a higher quality of life. Cronbach’s α for the original scale was 0.66 ~ 0.82. In this study, Cronbach’s α was 0.91.

### Parenting stress

The level of parental stress was measured using the Parental Stress Scale (PSS) by Berry and Jones [28]. The 18-item measure includes both positive, such as the emotional benefits and personal development, and negative, such as the demands on resources and restrictions, themes of parenthood. Each item was rated on a 5-point Likert scale (1 = “strongly disagree”, 2 = “disagree”, 3 = “undecided”, 4 = “agree”, 5 = “strongly agree”.) with high scores indicating a high level of stress. Regarding this scale’s reliability at the time of development, Cronbach’s α for the original scale was.83. In this study, Cronbach’s α was 0.62.

### Coping strategies

Coping level was measured using the 28-item coping orientation to problems experienced inventory (Brief-COPE) developed by Carver [17]. This instrument can be used to measure someone’s primary coping styles with scores on three subscales (Problem-focused coping, Emotion-focused coping, and Avoidant coping). This scale is rated on a 4-point Likert scale (1 = “I haven’t been doing this at all”, 2 = “A little bit”, 3 = “A medium amount”, 4 = “I’ve been doing this a lot). The highest scaled score shows which way of coping is frequently used. Cronbach’s α for the original scale was.50 or over. In this study, Cronbach’s α was 0.62 ~ 0.73.

### Semi-structured interview

Semi-structured interview questions were developed to explore parenting experiences, maternal quality of life, coping strategies, and formal and informal support systems. Table 1 presents the interview guide. To enhance the trustworthiness of the interview process, the authors and interpreters participated in all interviews and discussed interview procedures throughout the data collection process. Each interview lasted approximately one hour. Interviews were conducted in Swahili with the assistance of trained Tanzanian research assistants, who served as interpreters and facilitated culturally appropriate communication throughout the interviews. The interview questions were provided to participants in advance. All interviews were audio-recorded with participants’ consent, transcribed verbatim, translated into English for analysis, and the recordings were destroyed after transcription. Data collection continued until thematic saturation was achieved, defined as the point at which no substantially new themes emerged from subsequent interviews.

**Table 1:** Semi-structured interview questions.

Categories	Questions
General experiences in child-rearing	– Can you tell me about your daily routine as a mother? – What is the biggest joy and challenge in raising children in your community? – Has being a mother changed how you experience stress in your life?
Mothers’ subjective quality of life	– How satisfied are you with your life overall – Do you experience any health challenges that affect your daily life? – Do you feel supported by family, friends, or your community?
Coping Strategies	– When faced with a parenting problem, what do you usually do to try to solve it? – How do you manage your emotions when parenting becomes overwhelming?
Formal/informal support system for parents in child-rearing	– Are there any parenting support policies or services you currently receive? – Where do you get the information necessary for parenting? – Who takes care of your children when you are sick? – Do you and your spouse share the same views on your children’s education? – What do you think are the strengths and weaknesses of the current education system?
Hopes and Recommendations	– What do you think would improve your quality of life as a mother? – What kind of support would make parenting easier for you? – If you had the opportunity to advise decision-makers or community leaders, what changes would you suggest?

### Data analysis

#### Quantitative study.

The survey data were analyzed using SPSS Statistics 22.0 (IBM Corp., Armonk, NY, USA). Descriptive statistics were used for demographic characteristics, parental stress, coping styles, and quality of life. We used the Cronbach’s α coefficient to verify the internal reliability of the measures. We analyzed differences in the participants’ parental stress, coping styles, and quality of life according to participants’ general characteristics using independent t-tests and ANOVA. The correlations among the main variables were analyzed using Pearson’s correlation coefficients. The factors associated with maternal quality of life were analyzed using multiple linear regression analyses. The significance level was set at p < .05.

### Qualitative study

The transcribed interview data were coded using the qualitative research analysis program MAXQDA 2020 (VERBI Software, 2019). The analysis followed the six phases proposed by Braun and Clarke’s [29] thematic analysis method: familiarization with the data, generation of initial codes, searching for themes, reviewing themes, defining and naming themes, and producing the report. The first and second authors independently reviewed the transcripts and generated initial codes. Differences in coding and theme interpretation were discussed until consensus was reached. To enhance the trustworthiness of the interviews, the authors and interpreters participated in all interviews and discussed interview procedures throughout the data collection process. The qualitative findings were used to contextualize and explain the quantitative results, thereby enhancing the overall interpretation of factors associated with maternal quality of life. Integration occurred through the comparison of themes and quantitative findings to identify complementary and convergent evidence regarding factors associated with maternal quality of life.

## Results

### Demographic characteristics of quantitative research

The demographic characteristics of participants in the survey are summarized in Table 2. Participants’ average age was 36.65 ± 10.22 years. Regarding the number of children, the highest proportion (25.8%) was 2, while the smallest proportion (13.9%) was 5 or more. As for the child with disability of participants, a similar pattern appears for children with or without disabilities. Regarding marital status, most of the participants were married (58.1%). Among the education levels of participants, 8.1% had not completed primary school graduation, 46.2% had completed elementary school, 29.0% had completed high school, and 16.7% had completed college. Among the education levels of the fathers, the distribution was similar to that of participants: 9.7% had not completed primary school graduation, 45.2% had completed elementary school, 24.2% had completed high school, and 15.0% had completed college. As for income-generating activities, almost all participants came from a household with no (52.2%) or one (43.5%) income-generating activity. Among the income levels, the highest proportion (64.5%) was in the 5th quintile.

**Table 2:** General characteristics (*N* = 186).

Characteristics	Classification	Frequency (%)	M ± SD
Age (years)	20-24	14 (7.5)	36.65 ± 10.22
	25-29	36 (19.3)	
	30-34	39 (21.1)	
	35-39	35 (18.8)	
	Over 40	62 (33.3)	
Number of children	1	39 (21.0)	
	2	48 (25.8)	
	3	42 (22.6)	
	4	31 (16.7)	
	5 or more	26 (13.9)	
Child with disability	Yes	88 (47.3)	
	No	98 (52.7)	
Marital status	Single	53 (28.5)	
	Married	108 (58.1)	
	Separated/Divorced	12 (6.4)	
	Widowed	13 (7.0)	
Maternal education	No Education	15 (8.1)	
	Elementary	86 (46.2)	
	High	54 (29.0)	
	College	31 (16.7)	
Father’s education	No Education	18 (9.7)	
	Elementary	84 (45.2)	
	High	45 (24.2)	
	College	28 (15.0)	
	Father not present	11 (5.9)	
Income-generating activities	None	97 (52.2)	
	One activity	81 (43.5)	
	Two activities or more	8 (4.3)	
Wealth index, quintile	1st quintile (lowest)	2 (1.1)	
	2nd quintile	0 (0.0)	
	3rd quintile	14 (7.5)	
	4th quintile	50 (26.9)	
	5th quintile (highest)	120 (64.5)	

*SD*: standard deviation

### Parental stress, coping, and quality of life

The mean score for parental stress was 44.69 ± 8.87. In terms of coping styles, the mean scores for problem-focused coping, emotion-focused coping, and avoidant-focused coping were 3.15 ± 0.64, 2.80 ± 0.57, and 2.21 ± 0.59, respectively. The mean score for quality of life was 61.11 ± 11.91 (Table 3).

**Table 3:** Parental stress, coping styles, and quality of life.

Variables	M ± *SD*	Min	Max	Cronbach’s α
Parental stress	44.69 ± 8.87	22	64	0.63
Coping styles				
Problem-focused coping	3.15 ± 0.64	1	4	0.73
Emotion-focused coping	2.80 ± 0.57	1	4	0.72
Avoidant-focused coping	2.21 ± 0.59	1	4	0.62
Quality of life	61.11 ± 11.91	33.19	86.33	0.91

*SD*: standard deviation

### Differences in parental stress, coping, and quality of life according to general characteristics

We found significant differences in parental stress scores according to whether participants lived with children with a disability or not (t = 2.30, p = .023), marital status (F = 3.35, p = .020), fathers’ education levels (F = 2.67, p = .034), and wealth index (F = 6.43, p = .002). A post hoc analysis revealed that, for the maternal status, single participants had higher parental stress than did those who were married. As for the fathers’ education level, fathers with a college degree had lower parental stress scores than those with no education. Among income levels, participants in the 4th quintile had higher parental stress scores than did those in the 5th quintile. We also found differences in problem-focused coping scores according to age (F = 5.88, *p* = .003). The results of a post-hoc analysis revealed that participants aged 20–29 years had lower problem-focused coping than those aged 40 years and over. Avoidant-focused coping scores differed according to marital status (F = 3.93, *p* = .010). The results of a post-hoc analysis revealed that, for the maternal status, single participants had higher avoidant-focused coping scores than did those who were married. Quality of life scores differed according to wealth index (F = 4.92, *p* = .008). The results of a post-hoc analysis revealed that, as for the wealth index, participants in the 5th quintile had a higher quality of life score than did those in the 4th quintile (Table 4).

**Table 4:** Parental stress, coping, and quality of life according to general characteristics.

Characteristics	Parental stress			Problem-focused coping			Emotion-focused coping			Avoidant-focused coping			Quality of life		
	M ± SD	t/F	p	M ± SD	t/F	p	M ± SD	t/F	p	M ± SD	t/F	p	M ± SD	t/F	p
Age															
20-29^a^	45.50 ± 9.19	.34	.710	2.92 ± 0.68	5.88	.003	2.74 ± 0.59	.39	.677	2.30 ± 0.61	.83	.438	60.40 ± 11.36	.70	.498
30-39^b^	44.63 ± 8.55			3.16 ± 0.58		(a < c)	2.82 ± 0.56			2.19 ± 0.59			60.35 ± 12.44		
Over 40^c^	44.10 ± 9.09			3.32 ± 0.62			2.82 ± 0.58			2.16 ± 0.58			62.57 ± 11.74		
Number of children															
1	45.20 ± 8.51	1.16	.332	3.11 ± 0.59	2.06	.088	2.81 ± 0.42	1.37	.246	2.27 ± 0.53	1.00	.410	60.11 ± 11.43	1.03	.395
2	44.44 ± 10.19			3.16 ± 0.59			2.81 ± 0.54			2.27 ± 0.53			60.47 ± 13.27		
3	43.14 ± 8.94			3.00 ± 0.66			2.71 ± 0.63			2.10 ± 0.62			59.64 ± 11.20		
4	44.00 ± 7.42			3.14 ± 0.71			2.70 ± 0.61			2.10 ± 0.68			61.61 ± 12.76		
5 or more	47.69 ± 8.04			3.45 ± 0.62			3.01 ± 0.65			2.30 ± 0.61			62.20 ± 10.09		
Child with disability															
Yes	46.18 ± 7.84	2.30	.023	3.20 ± 0.62	.94	.349	2.84 ± 0.58	.85	.397	2.14 ± 0.53	−1.41	.160	59.95 ± 12.09	−1.14	.254
No	43.22 ± 9.47			3.11 ± 0.65			2.76 ± 0.57			2.27 ± 0.63			61.94 ± 11.63		
Marital status															
Single^a^	47.60 ± 7.93	3.35	.020	3.15 ± 0.64	.46	.713	2.82 ± 0.63	.17	.920	2.43 ± 0.53	3.93	.010	59.51 ± 11.32	2.39	.070
Married^b^	43.04 ± 8.90		(a > b)	3.13 ± 0.67			2.78 ± 0.58			2.10 ± 0.61		(a > b)	62.80 ± 11.88		
Separated/Divorced^c^	45.58 ± 10.66			3.16 ± 0.57			2.78 ± 0.40			2.18 ± 0.56			59.85 ± 12.11		
Widowed^d^	45.62 ± 8.21			3.35 ± 0.40			2.88 ± 0.42			2.24 ± 0.43			54.71 ± 12.49		
Maternal education															
No Education	46.60 ± 9.16	1.86	.139	3.16 ± 0.64	3.16	.026	2.66 ± 0.61	1.55	.204	2.05 ± 0.60	.47	.705	60.04 ± 14.46	.72	.541
Elementary	44.61 ± 8.77			3.23 ± 0.63			2.89 ± 0.59			2.23 ± 0.55			62.28 ± 11.50		
High	46.02 ± 9.05			2.94 ± 0.65			2.73 ± 0.55			2.23 ± 0.61			59.34 ± 11.82		
College	41.68 ± 8.32			3.30 ± 0.57			2.73 ± 0.52			2.17 ± 0.65			61.43 ± 12.05		
Father’s education															
No Education^a^	49.33 ± 8.09	2.67	.034	3.22 ± 0.61	.33	.860	2.69 ± 0.59	2.34	.057	2.26 ± 0.61	.71	.586	63.38 ± 13.15	.76	.552
Elementary^b^	45.18 ± 8.98		(a > d)	3.19 ± 0.60			2.91 ± 0.55			2.23 ± 0.59			60.64 ± 11.69		
High^c^	44.60 ± 8.24			3.08 ± 0.70			2.81 ± 0.60			2.25 ± 0.54			62.04 ± 11.98		
College^d^	41.04 ± 9.27			3.12 ± 0.69			2.62 ± 0.51			2.04 ± 0.68			61.51 ± 11.53		
Father not present^e^	43.09 ± 8.01			3.08 ± 0.61			2.53 ± 0.57			2.17 ± 0.49			56.07 ± 12.75		
Wealth index, quintile															
3rd quintile or lower^a^	48.31 ± 8.79	6.43	.002	2.97 ± 0.58	.82	.444	2.85 ± 0.65	.13	.880	2.27 ± 0.50	.10	.905	56.41 ± 13.40	4.92	.008
4th quintile	47.59 ± 6.63		(b > c)	3.20 ± 0.64			2.77 ± 0.49			2.22 ± 0.59			57.88 ± 10.10		(b < c)
5th quintile (highest)^c^	43.03 ± 9.28			3.15 ± 0.64			2.80 ± 0.59			2.20 ± 0.60			63.08 ± 12.02		

Abbreviations: SD, standard deviation.

a,b,c,d,e Groups with different superscript letters differ significantly according to Scheffé’s post hoc test.

### Relationships between parental stress, coping, and quality of life

The correlations between the major variables are shown in Table 5. Parental stress showed a significant positive correlation with emotion-focused coping (r = 0.19, p = .010), avoidant-focused coping (r = 0.38, p < .001), and quality of life (r = −0.15, p = .039). Problem-focused coping showed a significant positive correlation with emotion-focused coping (r = 0.64, p = < .001), avoidant-focused coping (r = 0.46, p = < .001), and quality of life (r = 0.35, p = < .001). Emotion-focused coping was observed positive correlation with avoidant-focused coping (r = 0.59, p = < .001) and quality of life (r = 0.37, p = < .001).

**Table 5:** Correlations between parental stress, coping, and quality of life.

Variable	Parental stress	Problem-focused coping	Emotion-focused coping	Avoidant-focused coping	Quality of life
Parental stress	1				
Problem-focused coping	0.16	1			
Emotion-focused coping	0.19 (.010)	0.64 (<.001)	1		
Avoidant-focused coping	0.38 (<.001)	0.46 (<.001)	0.59 (<.001)	1	
Quality of life	−0.15(.039)	0.35 (<.001)	0.37 (<.001)	0.07	1

### Factors associated with quality of life

Multiple regression analyses were conducted to examine the factors independently associated with quality of life. The variance inflation factors (VIFs) and tolerance values were examined to assess multicollinearity. The assumptions for the multiple regression analyses were satisfied.

Table 6 shows the results of the regression analyses, which were conducted with a total of 4 predictor variables. The regression model was found to be significant (F = 10.26, *p* < .001), and the adjusted coefficient of determination (Adj R2), which indicates the explanatory power of the model, was.233. The significant factors related to quality of life included problem-focused coping (B = 3.62, *p* = .027), emotion-focused coping (B = 8.17, *p*=<.001), and avoidant-focused coping (B = −3.99, *p* = .024). These together explained 23.3% of the variance in quality of life.

**Table 6:** Factors associated with quality of life.

Variables	Quality of life				
	B	SE	*β*	*t*	*p*
(Constant)	38.38	6.32		6.08	<.001
4th quintile (vs 3rd quintile or lower)	1.08	3.06	.04	.35	.724
5th quintile (vs 3rd quintile or lower)	5.40	2.84	.22	1.90	.059
Parental stress	−.15	.10	−.11	−1.46	.147
Problem-focused coping	3.62	1.63	.19	2.23	.027
Emotion-focused coping	8.17	1.97	.39	4.15	<.001
Avoidant-focused coping	−3.99	1.75	−.20	−2.28	.024

Adjusted R^2^ = .233, F (p) = 10.26 (<.001), VIF = 1.307 ~ 3.075.

Note: Wealth index, quintile was included as a control variable in all regression models.

### Content analysis of semi-structured interviews

Semi-structured interviews with nine local stakeholders revealed three overarching themes: parental stress as a daily struggle, adaptive and strained coping strategies, and structural and cultural barriers. These themes illustrate how mothers face persistent financial and emotional pressures, adopt both supportive and strained coping mechanisms, and navigate broader systemic challenges rooted in economic and social inequalities. Table 7 summarizes the main themes and associated sub-themes that emerged from the content analysis.

**Table 7:** Main themes and sub-themes were identified from the qualitative analysis.

Main Themes	Sub-themes
Parental Stress as a daily struggle	Financial issues Emotional costs of seeking support Erosion of parental self-efficacy Intergenerational stress transmission concerns
Adaptive and Strained Coping Strategies	Faith-based and peer support as sources of emotional resilience Exhaustion and helplessness under chronic stress
Structural and Cultural Barriers	Educational Trade-Offs due to economic opportunity costs Additional barriers for children with disabilities Perceived absence of fathers in parenting roles Lack of institutional or policy-level support

### Parental stress as a daily struggle

Under the theme of parental stress, participants frequently cited financial issues as a primary burden. One parent said, “The hardest part is the financial difficulties. It really requires a lot of money. Children don’t grow up on their own, you know” (P2). Teachers also demonstrated a clear understanding of these financial struggles. As one teacher explained, “I always emphasize the importance of education when speaking with parents, but that doesn’t mean I fail to understand the challenges they face” (PR2).

Parents also perceived emotional burdens associated with seeking help, as well as the unrelenting responsibility placed solely on mothers. One principal said, “Everyone is going through hard times. In such a situation, seeking help from the community requires considerable courage. There’s also a shared sense of obligation to repay any support received” (PR1). Another parent similarly noted, “It’s always the mother struggling alone to raise the child. Taking care of the child, arranging school, even finding someone to help—it’s all left to the mother. So of course, she has no energy left to plan or ask for support” (P1). In particular, parents raising children with disabilities feel a strong sense of isolation due to societal prejudice. One parent mentioned, “Everyone is struggling. We just vent to each other, but at some point, it feels like there’s no one left to share our struggles with. It’s lonely and tough” (P2).

A salient sub-theme was the erosion of parental self-efficacy, as some mothers expressed doubts about their parenting capacities. One mother shared, “Sometimes I wonder if I’m even doing a good job as a mother. No matter what I try, things don’t get better” (P1), “I feel like I’ve failed my children. I can’t give them what they need, emotionally or financially” (P1).

Additionally, several participants highlighted intergenerational stress transmission, noting how children absorb and reflect their parents’ distress. “My daughter asks me why I’m always sad. I try to hide it, but she sees everything” (T2). Another parent added, “I don’t want my children to grow up thinking this kind of struggle is normal, but I don’t know how to protect them from it” (P1). A parent with a child with a disability shared, “When I die, who will take care of my disabled child? It’ll probably fall to his siblings, and I already worry about passing this hardship on to them” (P2).

### Adaptive and strained coping strategies

In terms of coping strategies, participants shared both supportive and draining experiences. Faith-based and peer support—particularly within church communities—were cited as important sources of emotional strength, offering a sense of solidarity despite ongoing struggles. A local district commissioner remarked, “It seems like mothers often gather at church. Churches in the community play an important role in bringing people together and strengthening bonds. Maybe it’s because they share the same beliefs, but there’s a kind of unspoken understanding among them” (DC1). Several teachers and one parent echoed this sentiment. One shared, “When I pray or talk to other mothers in church, I feel lighter. It doesn’t solve the problem, but I feel stronger” (T1). Another added, “Church is the only place where I feel like I’m not alone. Other mothers are going through the same thing” (P1).

However, these coping efforts often occurred alongside feelings of helplessness and exhaustion stemming from chronic stress. While some adaptive strategies were present, there was also evidence of emotional withdrawal and resignation, suggesting limited access to sustained coping resources. One parent and a teacher explained, “We support each other, but everyone is tired. Sometimes it feels like we’re just surviving, not really living” (P1), “Even after talking to others, I still feel drained. Nothing really changes” (T3).

### Structural and cultural barriers

The third theme, structural and cultural barriers, encompassed broader systemic challenges. These included economic opportunity costs for children’s education, as parents often had to choose between income-generating activities and ensuring their children’s school attendance. Most respondents recognized the value of education and hoped that, through schooling, their children would secure good jobs and eventually contribute to the family’s finances. One principal shared, “I know how important education is. It’s very different from how our parents viewed education when I was in school. I understand that getting an education can lead to better jobs and, most importantly, the ability to earn more money” (PR2). However, economic hardships often made it difficult for parents to support their children’s schooling. One mother explained, “The school is far away, so I can’t take them to and from school every day. Sometimes I think it would be better to save that money and provide better meals. Also, if I get a small job, I have to rush to work, and it’s hard to supervise whether the kids are going to school or not” (P2). Limited resources also forced many to prioritize one child over another, leading to emotional distress and perceived inequity. As one teacher expressed as a mother, “I try to give each of my children the same chance, but sometimes I have to choose who goes to school and who stays home. It breaks my heart” (T1).

Additionally, parents raising children with disabilities faced added barriers due to social prejudice and a lack of understanding within the community. One mother taking care of a child with special need explained, “People say that even disabled children can go to school, but people in the neighborhood ask why I’m sending a disabled child to school. Honestly, I also don’t know what my child would be able to do at school” (P2). Stigma toward children with disabilities often extends to their family members, damaging their social reputation and reducing the support they receive from society. The mother shared, “Before I had a child with a disability, I was still able to do small jobs while raising my child. But after my child with a disability was born, both family and friends distanced themselves from me, and I couldn’t find any work. But what can I do? It’s my child, so I just have to raise them on my own as best as I can” (P2). While inclusive education is legally mandated in Tanzania, social stigma remains a significant barrier. One education officer noted, “The Tanzanian government is making significant efforts to promote inclusive education for children with disabilities. A lot of funding is indeed being allocated to train teachers in special education. But there are still many obstacles to putting these efforts into practice on the ground” (E1).

A notable cultural factor was the perceived absence of fathers in parenting, which placed disproportionate caregiving demands on mothers. “Their father thinks raising children is the mother’s job. He provides money sometimes, but he’s not really involved” (T2). Several participants described how fathers were physically present in the household but emotionally and practically detached from daily parenting responsibilities. This lack of engagement often left mothers feeling isolated and overwhelmed, with limited emotional or logistical support. As one respondent explained, “Even when he’s home, it’s like I’m parenting alone. He doesn’t know what the kids need or what they’re going through” (P1). Cultural norms were also seen as reinforcing this imbalance, with caregiving still widely regarded as a mother’s domain. One teacher further reflected, “We talk to the mothers at school, not the fathers. They rarely come to meetings or know how their children are doing. That’s what mothers are for” (T2). This dynamic raised concerns not only about maternal well-being but also about the lack of shared parenting as a model for children’s development.

Stakeholders also emphasized a lack of institutional or policy-level support, which reflects systemic neglect of caregiving families in low-income urban settings. Respondents reported that few formal or informal parenting support structures exist, leaving parents to shoulder the burden alone. A participant who was both a teacher and a parent said, “You do get help from various people when raising children. Sometimes I leave my child with a friend while I work, or ask the neighborhood kids to play with them. But ultimately, raising the child is in the hands of the mother. There’s no one to rely on” (T1). Another parent echoed this, saying, “It’s a mother’s role to raise the child. No aspect of a child’s development doesn’t require a mother’s involvement” (P2). A principal involved in special education added, “We’ve asked for help many times. Still, no one from the government ever follows up. We’re left to figure things out on our own” (PR1).

### Integration of quantitative and qualitative findings

The qualitative findings provided contextual explanations for the quantitative associations observed in this study and demonstrated how mothers’ daily experiences shaped their quality of life. First, although parental stress was negatively correlated with quality of life, this relationship was attenuated after accounting for coping strategies. Although the present cross-sectional design does not permit mediation testing, the findings suggest that coping processes may represent one pathway through which parenting-related stress influences maternal well-being. The negative association between parental stress and quality of life was reflected in participants’ descriptions of persistent financial hardship, emotional burdens, and concerns regarding the future of their children, particularly among mothers raising children with disabilities. These narratives illustrated how chronic parenting-related stress extends beyond individual psychological strain and is deeply embedded in socioeconomic challenges and caregiving responsibilities.

Second, the positive associations of problem-focused and emotion-focused coping with quality of life were supported by participants’ accounts of seeking emotional and practical support from church communities, peers, and social networks. These findings suggest that adaptive coping strategies may help mothers maintain psychological well-being despite ongoing adversities.

In contrast, the negative association between avoidant coping and quality of life was reflected in participants’ descriptions of emotional exhaustion, withdrawal, helplessness, and resignation. Several mothers reported feeling isolated and overwhelmed by caregiving demands and perceived a lack of meaningful support, suggesting that avoidant coping may further exacerbate psychological distress and reduce overall well-being.

Finally, the qualitative findings identified broader structural and cultural barriers—including poverty, social stigma toward disability, limited institutional support, and the perceived absence of fathers in parenting—that were not fully captured by the quantitative variables. These findings indicate that maternal quality of life in Tanzania is influenced not only by psychosocial factors but also by wider social and structural contexts. Together, the integration of quantitative and qualitative findings provides a more comprehensive understanding of maternal well-being and highlights the importance of addressing both individual coping resources and contextual barriers in efforts to improve maternal quality of life.

## Discussion

This study examined the relationships among parental stress, coping strategies, and maternal quality of life among mothers living in Kigamboni, Tanzania, using a convergent mixed-methods approach. The integrated findings demonstrate that both individual psychosocial factors and broader social and structural conditions, including economic hardship, limited parenting support systems, and social stigma toward disability, shape maternal quality of life. Quantitative analyses identified coping strategies as the strongest correlates of maternal quality of life, whereas qualitative findings explained how and why these relationships emerged within the everyday realities of mothers living in Tanzania.

### Psychosocial factors associated with maternal quality of life

This study revealed that mothers in Tanzania experience moderate to high levels of parental stress, with an average stress score comparable to findings from other sub-Saharan African contexts such as rural Zambia and Kenya [30]. Although parental stress showed a modest negative correlation with quality of life, it was no longer a significant independent predictor after accounting for coping strategies and socioeconomic factors. This finding suggests that the way mothers cope with chronic stress may be more important than the presence of stress itself [31]. The qualitative findings further illustrated how parenting stress was embedded in mothers’ everyday experiences of financial hardship, emotional isolation, and concerns about their children’s futures. Particularly among mothers raising children with disabilities, stress extended beyond immediate caregiving demands and reflected broader concerns regarding long-term caregiving responsibilities and social exclusion. These findings suggest that parenting stress should be understood not merely as an individual psychological burden but as a reflection of chronic socioeconomic and caregiving challenges that undermine maternal well-being. Consequently, interventions aimed at improving maternal quality of life should not only reduce chronic parenting-related stress but also strengthen mothers’ adaptive coping resources and the contextual supports that facilitate effective coping.

The findings also highlighted the important role of coping strategies in maternal well-being. Problem-focused and emotion-focused coping were positively associated with quality of life, whereas avoidant coping was negatively associated with well-being [32,33]. Qualitative findings help explain these relationships by showing that mothers frequently relied on faith-based communities, peer networks, and informal emotional support to cope with parenting challenges. These social resources appeared to provide emotional reassurance and practical support, potentially buffering the negative effects of chronic stress. In contrast, narratives describing emotional withdrawal, exhaustion, and feelings of helplessness reflected patterns consistent with avoidant coping and may contribute to reduced psychological well-being. These findings extend the Transactional Model of Stress and Coping by suggesting that the effectiveness of coping processes in low-resource settings may depend not only on individual appraisals and coping efforts but also on the availability of social and structural resources that support adaptive coping [14,15].

### Sociodemographic and structural vulnerabilities

Several sociodemographic factors were also associated with parental stress and coping. Parents of children with disabilities reported significantly higher stress levels, a finding that has been consistently reported in previous studies [34]. The qualitative findings suggest that these mothers experience a double burden in which intensive caregiving responsibilities coexist with social stigma and limited access to specialized services. These intersecting challenges may explain the substantially higher levels of parenting stress observed among these mothers and highlight the importance of addressing both practical support needs and broader societal attitudes toward disability.

Parental stress was also higher among mothers living in households where fathers had no formal education and among those in lower wealth quintiles. The association between lower paternal education, lower household wealth, and higher parental stress may reflect the cumulative disadvantages experienced by socioeconomically vulnerable families [3,4,6]. Fathers with limited education may experience fewer employment opportunities and reduced economic resources available for parenting. Qualitative findings further illustrated that many mothers perceived themselves as carrying parenting responsibilities with little practical support, suggesting that economic disadvantage and limited paternal involvement may jointly exacerbate maternal stress. Conversely, mothers in the highest wealth quintile reported significantly better quality of life, reinforcing previous evidence regarding the strong relationship between socioeconomic status and well-being [35].

Older mothers were more likely to engage in problem-focused coping, consistent with previous studies [36]. This finding suggests that coping strategies may evolve across the life course and that younger mothers may benefit from interventions designed to strengthen adaptive coping skills.

Single mothers engaged more frequently in avoidant coping than married mothers [37]. Qualitative narratives suggested that many single mothers experienced parenting as an isolated responsibility with limited opportunities for emotional recovery or shared decision-making. Under such circumstances, avoidant coping may reflect prolonged stress and limited coping resources. These findings identify single mothers as a particularly vulnerable group requiring targeted support.

The qualitative findings further revealed that maternal quality of life was shaped by broader structural and cultural conditions that were not fully captured by the quantitative analyses. Participants repeatedly described the absence of formal parenting support systems, economic hardship, social stigma toward disability, and limited paternal involvement in child-rearing. These findings suggest that maternal well-being in Tanzania cannot be fully understood solely through individual psychosocial characteristics because mothers’ experiences are strongly influenced by social and structural inequalities. The relatively modest explanatory power of the regression model may therefore reflect the multidimensional nature of maternal quality of life and the importance of contextual factors that were beyond the scope of the quantitative model.

The qualitative findings also revealed that educational systems play an important role not only in supporting children’s development but also in shaping maternal well-being. Participants frequently described school attendance as providing opportunities for mothers to engage in income-generating activities and temporarily alleviating caregiving demands. The findings suggest that access to education may represent an important contextual resource that can indirectly support maternal well-being by reducing economic pressure and caregiving demands [38]. However, mothers of children with disabilities reported that these potential benefits were often constrained by inadequate educational services, weak informal support networks, and persistent social stigma. These mothers experienced a double burden in which intensive caregiving responsibilities coexisted with social exclusion and limited access to resources. Such experiences may help explain the substantially higher levels of parenting stress observed among parents of children with disabilities in the quantitative analyses. Similar findings have been reported in previous studies showing that parents of children with disabilities frequently experience social marginalization and reduced access to community support [39].

### Practical implications

The findings have several practical implications. First, community-based parenting support programs that strengthen peer networks and informal social support may enhance mothers’ adaptive coping capacities and resilience. Because churches were frequently identified as trusted sources of emotional and practical support in the qualitative interviews, faith-based organizations may serve as valuable partners in delivering parenting support interventions. Second, targeted services for mothers raising children with disabilities are needed to address both caregiving burdens and social stigma. Third, policies that promote fathers’ involvement in parenting and expand access to formal parenting support services may reduce the disproportionate caregiving responsibilities currently borne by mothers in Tanzania. Finally, strengthening educational opportunities and economic support for vulnerable families may indirectly improve maternal quality of life by reducing chronic parenting-related stress.

### Limitations

This study has several limitations that should be considered when interpreting the findings. First, the cross-sectional design precludes causal inferences regarding the relationships among parental stress, coping strategies, and maternal quality of life. Longitudinal studies are needed to examine how these relationships evolve over time. Second, participants were recruited using convenience sampling from a limited geographic area in Kigamboni, Tanzania. Therefore, the findings may not be generalizable to all mothers in Tanzania or other low-resource settings. Third, all quantitative measures relied on self-reported data and may therefore be subject to recall bias and social desirability bias. Fourth, although parental stress and coping strategies significantly explained maternal quality of life, the explanatory power of the regression model was modest, suggesting that additional social, cultural, and community-level factors influencing maternal well-being were not captured in the present study. Future research should incorporate broader structural variables, including social support, access to services, and community resources.

Fifth, the internal consistency of the Parental Stress Scale (Cronbach’s α = 0.63) and the Avoidant-focused coping scale (Cronbach’s α = 0.62) was relatively modest, which may have affected the consistency of the measurements. Therefore, the findings should be interpreted with caution. Future studies are needed to further examine the psychometric properties of the Swahili versions of these instruments and to establish their reliability and validity in the Tanzanian population.

Finally, the qualitative component involved a relatively small number of purposively selected stakeholders and was intended to provide contextual understanding rather than statistical representation. Although thematic saturation was achieved, the transferability of the qualitative findings to other settings may be limited. Despite these limitations, the mixed-methods approach provided a comprehensive understanding of maternal well-being by integrating statistical associations with the lived experiences of mothers and community stakeholders in Tanzania.

## Conclusion

This mixed-methods study demonstrates that maternal quality of life in Tanzania is shaped by the interplay between psychosocial factors and broader social and structural conditions. Adaptive and maladaptive coping strategies emerged as the primary factors associated with maternal quality of life. Although parental stress was negatively correlated with quality of life, its effect appeared to operate through coping processes and broader contextual conditions, whereas adaptive coping strategies and supportive social resources appeared to promote maternal well-being. Qualitative findings further revealed that economic hardship, limited parenting support systems, social stigma toward disability, and unequal caregiving responsibilities influence how mothers experience and manage parenting-related stress. These findings underscore the importance of developing multilevel interventions that strengthen adaptive coping resources while simultaneously addressing the social and structural conditions that shape mothers’ capacity to cope with chronic parenting-related stress.

## Supporting information

S1 File Questionnaire (English and Swahili). The survey questionnaire used in this study.
(DOCX)

S2 File Inclusivity in Global Research Questionnaire. Completed PLOS ONE inclusivity in global research questionnaire.
(DOCX)
