# Supplementary material for: Parental stress, coping strategies, and maternal quality of life in urban Tanzania: A mixed-methods study
Source: PLoS One. 2026 Sep 25;21(9):e0358956. doi: 10.1371/journal.pone.0358956 (PMC13614557; doi:10.1371/journal.pone.0358956)
Supplement: Supplementary file 1 [file pone.0358956.s001.docx]

Questionnaires

1. What is your year of birth? (	)

2. What is the highest education you receive?

① None at all ② Elementary School ③ High School ④ College

3. Marital status? ① Single ② Married ③ Divorced/Separated ④ Widow or widower

4. Are you currently ill? ① Yes ② No

4-1. If something is wrong with your health, what do you think it is?

(	) illness/problem

5. Father’s education? ① None at all ② Elementary School ③ High School ④ College ⑤ No father

6. Income generating activities? ① None ② One activity ③ Two activities ④ Three activities

7. Does your household have…Electricity? ① Yes ② No

8. …A television? ① Yes ② No

9. …A radio? ① Yes ② No

10. …An iron? ① Yes ② No

11. Does any member of this household have a bank account? ① Yes ② No

12. What is the main material of the floor of your dwelling?

① Earth/Sand/Dung ② Cement/Concrete ③ Other (	)

13. What is the main material of the exterior walls of your dwelling?

① Cement Blocks ② Other (	)

14. What is the main material of the roof of your dwelling?

① Iron Sheet ② Grass/Thatch/Palm Leaf/Mud ③ Other (	)

15. What type of fuel does your household mainly use for cooking?

① Firewood ② Charcoal ③ Other (	)

16. What is the main source of energy for lighting in the household?

① Electricity ② Battery/Solar powered Flashlight or Lamp ③ Other (	)

17. Number of children? (	)

18. Do you have a child or children with disability? ① Yes ② No

18-1. If you have a child(ren) with disability, what kind of the handicapped?

1. seeing ② remembering ③ hearing ④ communication ⑤ walking ⑥ self-care ⑦Albinism ⑧ others (	)

| **The following questions ask about your LAST-BORN child or Child with Disability** | |
| --- | --- |
| 1. How old is your child? | Age (In completed years) ( ) |
| 2. Has ever attended school or any early childhood education programme? | ① Yes ② No |
| 3. At any time during the current school year did attend school or any early childhood education programme? | ① Yes ② No |
| 4. During this current school year, which level and grade or year is attending? | 1. Primary education 2. Secondary education 3. High school education |
| 5**.** Is your child covered by any health insurance? | ① Yes ② No |

Community member’s or parent’s views on children with disability

| Community member’s and Parents’ views | Strongly Agree or Important | Sometimes Agree or Somewhat Important | Strongly Disagree or Not Important |
| --- | --- | --- | --- |
| 1) Do you think that God has given you this child as a test, or that the goal of this experience is that you learn the true meaning of being a parent? | ① | ② | ③ |
| 2) Do you think that your child can get sick easier than other children? | ① | ② | ③ |
| 3) Do you think that your child can get hurt easier than other children can? | ① | ② | ③ |
| 4) How important is it your child does not struggle to do things that are difficult? | ① | ② | ③ |
| 5) How important is it to you that your child uses his or her braces and does his or her exercises? | ① | ② | ③ |
| 6) How important is it for you that your child be independent in dressing, feeding, mobility, and skills of communication? | ① | ② | ③ |
| 7) Is your child very attached to you? | ① | ② | ③ |
| 8) Is it important to give your child with a disability opportunities to do things that other children usually don’t get to do (go to concerts or special shows, etc), or give them things that the other children don’t usually get to have (special toys, bicycles, etc)? | ① | ② | ③ |
| 9) How important is it to you look for treatments that will make your child as independent as possible? | ① | ② | ③ |

Parental Stress Scale

| No. | Question | Strongly disagree | Disagree | Undecided | Agree | Strongly agree |
| --- | --- | --- | --- | --- | --- | --- |
| 1 | I am happy in my role as a parent | ① | ② | ③ | ④ | ⑤ |
| 2 | There is little of nothing I wouldn’t do for my child(ren) if it was necessary | ① | ② | ③ | ④ | ⑤ |
| 3 | Caring for my child(ren) sometimes takes more time and energy than I have to give | ① | ② | ③ | ④ | ⑤ |
| 4 | I sometimes worry whether I am doing enough for my child(ren) | ① | ② | ③ | ④ | ⑤ |
| 5 | I feel close to my child(ren) | ① | ② | ③ | ④ | ⑤ |
| 6 | I enjoy spending time with my child(ren) | ① | ② | ③ | ④ | ⑤ |
| 7 | My child(ren) is an important source of affection for me | ① | ② | ③ | ④ | ⑤ |
| 8 | Having child(ren) gives me a more certain and optimistic view for the future | ① | ② | ③ | ④ | ⑤ |
| 9 | The major source of stress in my life is my child(ren) | ① | ② | ③ | ④ | ⑤ |
| 10 | Having child(ren) leaves little time and flexibility in my life | ① | ② | ③ | ④ | ⑤ |
| 11 | Having child(ren) has been a financial burden | ① | ② | ③ | ④ | ⑤ |
| 12 | It is difficult to balance different responsibilities because of my child(ren) | ① | ② | ③ | ④ | ⑤ |
| 13 | The behavior of my child(ren) is often embarrassing or stressful to me | ① | ② | ③ | ④ | ⑤ |
| 14 | If I had it to do over again, I might decide not to have child(ren) | ① | ② | ③ | ④ | ⑤ |
| 15 | I feel overwhelmed by the responsibility of being a parent | ① | ② | ③ | ④ | ⑤ |
| 16 | Having child(ren) has meant having too few choices and too little control over my life | ① | ② | ③ | ④ | ⑤ |
| 17 | I am satisfied as a parent | ① | ② | ③ | ④ | ⑤ |
| 18 | I find my child(ren) enjoyable | ① | ② |  |  |  |

Brief-COPE

| No. | Question | I haven’t been doing this at all | A little bit | A medium amount | I’ve been doing this a lot |
| --- | --- | --- | --- | --- | --- |
| 1 | I've been turning to work or other activities to take my mind off things | ① |  |  | ④ |
| 2 | I've been concentrating my efforts on doing something about the situation I'm in | ① |  |  | ④ |
| 3 | I've been saying to myself "this isn't real" | ① |  |  | ④ |
| 4 | I've been using alcohol or other drugs to make myself feel better | ① |  |  | ④ |
| 5 | I've been getting emotional support from others | ① |  |  | ④ |
| 6 | I've been giving up trying to deal with it | ① |  |  | ④ |
| 7 | I've been taking action to try to make the situation better | ① |  |  | ④ |
| 8 | I've been refusing to believe that it has happened | ① | ② |  | ④ |
| 9 | I've been saying things to let my unpleasant feelings escape | ① | ② | ③ | ④ |
| 10 | I’ve been getting help and advice from other people | ① | ② | ③ | ④ |
| 11 | I've been using alcohol or other drugs to help me get through it | ① | ② | ③ | ④ |
| 12 | I've been trying to see it in a different light, to make it seem more positive | ① | ② | ③ | ④ |
| 13 | I’ve been criticizing myself | ① | ② | ③ | ④ |
| 14 | I've been trying to come up with a strategy about what to do | ① | ② | ③ | ④ |
| 15 | I've been getting comfort and understanding from someone | ① | ② | ③ | ④ |
| 16 | I've been giving up the attempt to cope | ① | ② | ③ | ④ |
| 17 | I've been looking for something good in what is happening | ① | ② | ③ | ④ |
| 18 | I've been making jokes about it | ① | ② | ③ | ④ |
| 19 | I've been doing something to think about it less, such as going to movies, watching TV,  reading, daydreaming, sleeping, or shopping | ① | ② | ③ | ④ |
| 20 | I've been accepting the reality of the fact that it has happened | ① | ② | ③ | ④ |
| 21 | I've been expressing my negative feelings | ① | ② | ③ | ④ |
| 22 | I've been trying to find comfort in my religion or spiritual beliefs | ① | ② | ③ | ④ |
| 23 | I’ve been trying to get advice or help from other people about what to do | ① | ② | ③ | ④ |
| 24 | I've been learning to live with it | ① | ② | ③ | ④ |
| 25 | I've been thinking hard about what steps to take | ① | ② | ③ | ④ |
| 26 | I’ve been blaming myself for things that happened | ① | ② | ③ | ④ |
| 27 | I've been praying or meditating | ① | ② | ③ | ④ |
| 28 | I've been making fun of the situation | ① | ② | ③ | ④ |

Quality of life

| No. | Question | Very Poor | Poor | Neither poor nor good | Good | Very good |
| --- | --- | --- | --- | --- | --- | --- |
| 1 | How would you rate your quality of life? | ① | ② | ③ | ④ | ⑤ |
|  |  | Very dissatisfied | Dissatisfied | Neither satisfied nor dissatisfied | Satisfied | Very satisfied |
| 2 | How satisfied are you with your health? | ① | ② | ③ | ④ | ⑤ |
| The following questions ask about how much you have experienced certain things in the last two weeks | | | | | | |
|  |  | Not at all | A little | A moderate amount | Very much | An extreme amount |
| 3 | To what extent do you feel that physical pain prevents you from doing what you need to do? | ① | ② | ③ | ④ | ⑤ |
| 4 | How much do you need any medical treatment to function in your daily life? | ① | ② | ③ | ④ | ⑤ |
| 5 | How much do you enjoy life? | ① | ② | ③ | ④ | ⑤ |
| 6 | To what extent do you feel your life to be meaningful? | ① | ② | ③ | ④ | ⑤ |
|  |  | Not at all | A little | A moderate amount | Very much | Extremely |
| 7 | How well are you able to concentrate? | ① | ② | ③ | ④ | ⑤ |
| 8 | How safe do you feel in your daily life? | ① | ② | ③ | ④ | ⑤ |
| 9 | How healthy is your physical environment? | ① | ② | ③ | ④ | ⑤ |
| The following questions ask about how completely you experience or were able to do certain things in the last two weeks | | | | | | |
|  |  | Not at all | A little | Moderately | Mostly | Completely |
| 10 | Do you have enough energy for everyday life? | ① | ② | ③ | ④ | ⑤ |
| 11 | Are you able to accept your bodily appearance? | ① | ② | ③ | ④ | ⑤ |
| 12 | Have you enough money to meet your needs? | ① | ② | ③ | ④ | ⑤ |
| 13 | How available to you is the information that you need in your day to day life? | ① | ② | ③ | ④ | ⑤ |
| 14 | To what extent do you have the opportunity for leisure activities? | ① | ② | ③ | ④ | ⑤ |
|  |  | Very Poor | Poor | Neither poor nor good | Good | Very good |
| 15 | How well are you able to get around? | ① | ② | ③ | ④ | ⑤ |

| The following questions ask you to say how good or satisfied you have felt about various aspects of your life over the last two weeks | | | | | | |
| --- | --- | --- | --- | --- | --- | --- |
| 16 | How satisfied are you with your sleep? | ① | ② | ③ | ④ | ⑤ |
| 17 | How satisfied are you with your ability to perform your daily living activities? | ① | ② | ③ | ④ | ⑤ |
| 18 | How satisfied are you with your capacity for work? | ① | ② | ③ | ④ | ⑤ |
| 19 | How satisfied are you with yourself? | ① | ② | ③ | ④ | ⑤ |
| 20 | How satisfied are you with your personal relationships? | ① | ② | ③ | ④ | ⑤ |
| 21 | How satisfied are you with your sex life? | ① | ② | ③ | ④ | ⑤ |
| 22 | How satisfied are you with the support you get from your friends? | ① | ② | ③ | ④ | ⑤ |
| 23 | How satisfied are you with the conditions of your living place? | ① | ② | ③ | ④ | ⑤ |
| 24 | How satisfied are you with your access to health services? | ① | ② | ③ | ④ | ⑤ |
| 25 | How satisfied are you with your transport? | ① | ② | ③ | ④ | ⑤ |
| The following question refers to how often you have felt or experienced certain things in the last two weeks | | | | | | |
|  |  | Never | Seldom | Quite often | Very Often | Always |
| 26 | How often do you have negative feelings such as blue mood, despair, anxiety, depression? | ① | ② |  |  |  |

Attitude on People with a disability

| No. | Question: Please indicate whether you agree or disagree with the following statements. | Strongly disagree | Disagree | Agree | Strongly agree |
| --- | --- | --- | --- | --- | --- |
| 1 | I do not want people with a disability living in my neighborhood. | ① | ② |  |  |
| 2 | People with disability should date and marry each other, regardless of whether his/her spouse or partner has a disability or not. | ① | ② |  |  |
| 3 | Most people with a disability are unpredictable and express impulsive behaviors. | ① | ② |  |  |
| 4 | People with a disability should be prevented from having children. | ① |  |  |  |

Parenting Practices

| No. | Question | Answer |
| --- | --- | --- |
| 1 | Excluding school text books and holy books, how many books do you have for (your LAST-BORN child or child with disability) to read at home? | Number of books   1. none 2. 1~3 books 3. 4~6 books 4. 7~9 books 5. 10 or more books |
| 2 | I am interested in learning about the things that (your LAST-BORN child or child with disability) plays with when (he/she) is at home  Does (he/she) play with: | |
|  | 2-1. Homemade toys, such as dolls, cars, or other toys made at home? | 1. YES 2. NO |
|  | 2-2. Toys from a shop or manufactured toys? | 1. YES 2. NO |
|  | 2.3. Household objects, such as bowls or pots, or objects found outside, such as sticks, rocks, animal shells or leaves? | 1. YES 2. NO |
| 3 | Sometimes adults taking care of children have to leave the house to go shopping, wash clothes, or for other reasons and have to leave young children.  3-1. On how many days left alone for more than an hour in the past week was (your LAST-BORN child or child with disability): | 1. none 2. 1 day 3. 2~3 days 4. 4~5 days 5. 5~7 days |
|  | 3-2. Left in the care of another child, that is, someone less than 10 years old, for more than an hour? | 1. none 2. 1 day 3. 2~3 days 4. 4~5 days 5. 5~7 days |

Child Discipline

| No. | Question | Answer |
| --- | --- | --- |
| 1 | **1**. Adults use certain ways to teach children the right behavior or to address a behavior problem. I will read various methods that are used. Please tell me if you or any other adult in your household has used this method with *(*your LAST-BORN child*)* in the past month. | |
|  | [A] Took away privileges, forbade something *(*your LAST-BORN child*)* liked or did not allow (him/her) to leave the house. | ① Yes ② No |
|  | [B] Explained why *(*your LAST-BORN child*)*’s behavior was wrong. | ① Yes ② No |
|  | [C] Shook (him/her). | ① Yes ② No |
|  | [D] Shouted, yelled at or screamed at (him/her). | ① Yes ② No |
|  | [E] Gave (him/her) something else to do. | ① Yes ② No |
|  | [F] Spanked, hit or slapped (him/her) on the bottom with bare hand. | ① Yes ② No |
|  | [G] Hit (him/her) on the bottom or elsewhere on the body with something like a belt, hairbrush, stick or other hard object. | ① Yes ② No |
|  | [H] Called (him/her) dumb, lazy or another name like that. | ① Yes ② No |
|  | [I] Hit or slapped (him/her) on the face, head or ears. | ① Yes ② No |
|  | [J] Hit or slapped (him/her) on the hand, arm, or leg. | ① Yes ② No |
|  | [K] Beat (him/her) up, that is hit (him/her) over and over as hard as one could. | ① Yes ② No |
| 2 | **2**. Do you believe that in order to bring up, raise, or educate a child properly, the child needs to be physically punished? | ① Yes ② No |

**Assess of health care services**

What limits do you have accessing health care services for your child?

| Items | No  problem | Some  problem | Moderate problem | Serious problem | Insurmountable problem |
| --- | --- | --- | --- | --- | --- |
| Lack of transport home to facility | ① | ② | ③ | ④ | ⑤ |
| Inadequate drugs or equipment | ① | ② | ③ | ④ | ⑤ |
| No services available | ① | ② | ③ | ④ | ⑤ |
| Journey dangerous | ① | ② | ③ | ④ | ⑤ |
| Couldn’t afford transport cost | ① | ② | ③ | ④ | ⑤ |
| Standard of the health facility | ① | ② | ③ | ④ | ⑤ |
| Physical access to facility | ① | ② | ③ | ④ | ⑤ |
| Don’t afford the cost | ① | ② | ③ | ④ | ⑤ |
| Not sick enough | ① | ② | ③ | ④ | ⑤ |
| Negative attitudes health workers | ① | ② | ③ | ④ | ⑤ |
| Previously badly treated | ① | ② | ③ | ④ | ⑤ |
| Tried but denied | ① | ② | ③ | ④ | ⑤ |
| Because of faith | ① | ② | ③ | ④ | ⑤ |
| Communication with health workers | ① | ② | ③ | ④ | ⑤ |
| No facility accommodation | ① | ② | ③ | ④ | ⑤ |
| Not knowing where to go | ① | ② | ③ | ④ | ⑤ |
| Couldn’t get time off work | ① | ② | ③ | ④ | ⑤ |
| Don’t have necessary document | ① | ② | ③ | ④ | ⑤ |

**Functioning with Disability**

If you have a child with a disability, how much difficulty does your child have doing these activities?

| Category | | No difficulty | Some difficulty | A lot of difficulty | Cannot do at all | *Refused* | *Don’t know* |
| --- | --- | --- | --- | --- | --- | --- | --- |
| Seeing | | ① | ② | ③ | ④ | ⑦ | ⑨ |
| Hearing | | ① | ② | ③ | ④ | ⑦ | ⑨ |
| Walking | | ① | ② | ③ | ④ | ⑦ | ⑨ |
| Communication in using usual language | | ① | ② | ③ | ④ | ⑦ | ⑨ |
| Cognition (such as remembering or concentration) | | ① | ② | ③ | ④ | ⑦ | ⑨ |
| Self-care (such as washing all over or dressing) | | ① | ② | ③ | ④ | ⑦ | ⑨ |
| Upper body | Raising a 2 liter bottle of water from waist to eye level | ① | ② | ③ | ④ | ⑦ | ⑨ |
|  | Using hands and fingers (such as picking up small objects) | ① | ② | ③ | ④ | ⑦ | ⑨ |
| Anxiety | How often do you feel worried, nervous or anxious? | ① Daily  ② Weekly  ③ Monthly  ④ A few times a year  ⑤ Never | | | | ⑦ | ⑨ |
|  | Thinking about the last time you felt worried, nervous or anxious, how would you describe the level of these feelings? | ① A little  ② A lot  ③ Somewhere in between a little and a lot | | | | ⑦ | ⑨ |
| Depression | How often do you feel depressed? | ① Daily  ② Weekly  ③ Monthly  ④ A few times a year  ⑤ Never | | | | ⑦ | ⑨ |
|  | Thinking about the last time you felt depressed, how depressed did you feel? | ① A little  ② A lot  ③ Somewhere in between a little and a lot | | | | ⑦ | ⑨ |

DODOSO

1. Umezaliwa mwaka gani? (	)

2. Elimu ya juu uliopata ni kiwago gani?

(a) Sijasoma kabisa (b) Shule ya Msingi (c) Shule ya Sekondari (d) Chuo

3. Hali ya ndoa?

(a) Sina ndoa (b) Ndoa (c) Talaka /Kutengana (d) mjane/ mgane

4. Je, kwa sasa unaumwa? (a) Ndiyo (b) Hapana

4-1. Kama una tatizo lolote kiafya, unadhani ni nini ? (	)

5. Kiwango cha elimu ya baba ni kipi?

(a) Hakusoma kabisa (b) Shule ya Msingi (c) Shule ya Sekondari (d) Chuo (5) Sina baba

6. Una shughuli gani za kuzalisha kipato?

(a) Sina (b) Shughuli moja (c) Shughuli mbili (d) Shughuli tatu

7. Je, kaya yako ina... Umeme? (a) Ndiyo (b) Hapana

8. Je, kaya yako ina	Televisheni? (a) Ndiyo (b) Hapana

9. Je, kaya yako ina	Redio? (a) Ndiyo (b) Hapana

10. Je, kaya yako ina	Pasi? (a) Ndiyo (b) Hapana

11. Je, mkazi yeyote wa kaya hii ana akaunti ya benki? (1) Ndiyo (2) Hapana

12. Ni nyenzo gani kuu ya sakafu ya makazi yako?

(a) Ardhi/Mchanga (b) Saruji (3) Nyingine (	)

13. Ni nyenzo gani kuu ya kuta za nje za makazi yako?

(a) ukuta wa Saruji (b) Vingine (	)

14. Ni nyenzo gani kuu ya paa la makazi yako?

(a) mabati (b) Nyasi/ makuti /nyasi/tope (c) Nyingine (	)

15. Kaya yako inatumia nini hasa kwa ajili ya kupikia?

(a) Kuni (b) Mkaa (c) Nyingine (	)

16. Nini chanzo kikuu cha nishati ya taa katika kaya yako?

(a) Umeme (b) Betri/ Taa za sola (c) Nyingine (	)

17. Una jumla ya watoto wangapi? (	)

18. Je, una mtoto au watoto wenye ulemavu? (a) Ndiyo (b) Hapana

18-1. Kama una mtoto (wa) mwenye ulemavu, ni aina gani ya ulemavu?

(a) kuona (b) kukumbuka (c) kusikia (d) mawasiliano (e) kutembea (f) kujitunza (g)Ualbino (h) nyingine (	)

| **Maswali yafuatayo yanauliza kuhusu mtoto wako wa MWISHO au Mtoto mwenye Ulemavu** | |
| --- | --- |
| 1. Mtoto wako ana umri gani? | Umri kwa miaka kamili ( ) |
| 2. Amewahi kuhudhuria shule au mpango wowote wa elimu ya awali? | ① Ndiyo ② Hapana |
| 3. Wakati wowote katika mwaka huu wa masomo alihudhuria shule au mpango wowote wa elimu ya awali? | ① Ndiyo ② Hapana |
| 4. Katika mwaka huu wa masomo , ni kiwango gani au daraja gani unahudhuria? | 1. Elimu ya msingi 2. Elimu ya sekondari 3. Elimu ya sekondari ya juu |
| 5**.** Je, mtoto wako anahudumiwa na bima yoyote ya afya? | ① Ndiyo ② Hapana |

Maoni ya mwanajamii au mzazi kuhusu watoto wenye ulemavu

| Maoni ya mwanajamii na wazazi | nakubaliana au muhimu | Wakati mwingine nakubaliana au muhimu kwa kiasi fulani | Sikubaliani nayo au sio muhimu |
| --- | --- | --- | --- |
| 1) Unadhani Mungu amekupa mtoto huyu kama mtihani, au kwamba lengo la uzoefu huu ni kwamba ujifunze maana halisi ya kuwa mzazi? | ① | ② | ③ |
| 2) Je, unadhani mtoto wako anaweza kuugua kwa urahisi kuliko watoto wengine? | ① | ② | ③ |
| 3) Je, unadhani mtoto wako anaweza kuumia kirahisi kuliko watoto wengine? | ① | ② | ③ |
| 4) Kuna umuhimu gani mtoto wako hajitahidi kufanya mambo ambayo ni magumu? | ① | ② | ③ |
| 5) Kuna umuhimu gani kwako kwamba mtoto wako anatumia nyenzo zake na anafanya mazoezi yake? | ① | ② | ③ |
| 6) Kuna umuhimu gani kwako kwamba mtoto wako awe huru katika kuvaa, kulisha, kuhama, na ujuzi wa mawasiliano? | ① | ② | ③ |
| 7) Je, mtoto wako amekuzoea sana na wewe? | ① | ② | ③ |
| 8) Je, ni muhimu kumpa mtoto wako fursa ya ulemavu kufanya vitu ambavyo kwa kawaida watoto wengine huwa hawapati kufanya (kwenda kwenye matamasha au maonyesho maalum, n.k), au kuwapa vitu ambavyo watoto wengine huwa hawapati kuwa navyo (midoli maalum, baiskeli, n.k)? | ① | ② | ③ |
| 9) Kuna umuhimu gani kwako kutafuta matibabu ambayo yatamfanya mtoto wako awe huru iwezekanavyo? | ① | ② | ③ |

Kiwango cha Msongo wa Mawazo ya Wazazi

| No. | Swali | Nakataa vikali | Nakataa | Sijaamua | Nkubali | Nakubali vikali |
| --- | --- | --- | --- | --- | --- | --- |
| 1 | Ninaifurahia nafasi yangu kama mzazi | ① | ② | ③ |  |  |
| 2 | Hakuna kitu ambachosiwezi fanya kwa ajili ya watoto wangu | ① | ② | ③ | ④ |  |
| 3 | Kuhudumia mtoto/watoto wangu wakati mwingine huchukua muda na nguvu zaidi kuliko ninavyopaswa kutoa | ① | ② | ③ | ④ | ⑤ |
| 4 | Wakati mwingine nina wasiwasi kama ninafanya vya kutosha kwa mtoto/ watoto wangu | ① | ② | ③ | ④ | ⑤ |
| 5 | Niko karibu na wanangu? mwanangu | ① | ② | ③ | ④ | ⑤ |
| 6 | Ninafurahi kutumia muda pamoja na wanangu | ① | ② | ③ | ④ | ⑤ |
| 7 | Mtoto/ watoto wangu ni chanzo muhimu cha mapenzi kwangu | ① | ② | ③ | ④ | ⑤ |
| 8 | Kuwa na mtoto/ watoto kunanipa mtazamo wa uhakika zaidi na wenye matumaini kwa siku zijazo | ① | ② | ③ | ④ | ⑤ |
| 9 | Chanzo kikubwa cha msongo wa mawazo katika maisha yangu ni mtoto/ watoto wangu | ① | ② | ③ | ④ | ⑤ |
| 10 | Kua na mtoto (watoto) imeacha muda kidogo na katika maisha yangu | ① | ② | ③ | ④ | ⑤ |
| 11 | Kupata mtoto/ watoto imekuwa mzigo wa kifedha | ① | ② | ③ | ④ | ⑤ |
| 12 | Ni vigumu kuweka usawa wa majukumu mbalimbali kwa sababu ya mtoto/ watoto wangu | ① | ② | ③ | ④ | ⑤ |
| 13 | Tabia ya mtoto/ watoto wangu mara nyingi huwa inanitia aibu au kunisumbua | ① | ② | ③ | ④ | ⑤ |
| 14 | Kama ningekuwa na nafasi ya kurudi tena, ningeamua kutokuwa na mtoto | ① | ② | ③ | ④ | ⑤ |
| 15 | Nahisi kuzidiwa na jukumu la kuwa mzazi | ① | ② | ③ | ④ | ⑤ |
| 16 | Kuwa na watoto kumesababisha kuwa na chaguo chache sana na udhibiti mdogo sana juu ya maisha yangu | ① | ② | ③ | ④ | ⑤ |
| 17 | Nimeridhika kama mzazi | ① | ② | ③ | ④ | ⑤ |
| 18 | Nawafurahia wanangu | ① | ② | ③ | ④ | ⑤ |

Brief COPE

| No. | Swali | Sijawahi kufanya | kidogo | Kiasi | Nimekua nikifanya hivi |
| --- | --- | --- | --- | --- | --- |
| 1 | Nimekuwa nikifanya kazi au shughuli nyingine ili kuondoa akili yangu kwenye mambo mengine | ① | ② | ③ | ④ |
| 2 | Nimekuwa nikielekeza juhudi zangu katika kufanya jambo fulani kuhusu hali niliyonayo | ① | ② | ③ | ④ |
| 3 | Nimekuwa nikijisemea moyoni "hii sio halisi" | ① | ② | ③ | ④ |
| 4 | Nimekuwa nikitumia pombe au madawa mengine kujifanya nijisikie vizuri | ① | ② | ③ | ④ |
| 5 | Nimekuwa nikipata msaada wa kihisia kutoka kwa wengine | ① | ② | ③ | ④ |
| 6 | Nimekuwa nikikata tamaa kujaribu kukabiliana nayo | ① | ② | ③ | ④ |
| 7 | Nimekuwa nikichukua hatua kujaribu kufanya hali iwe nzuri zaidi | ① | ② | ③ | ④ |
| 8 | Nimekuwa nikikataa kuamini kuwa imetokea | ① | ② | ③ | ④ |
| 9 | Nimekuwa nikisema mambo ili hisia zangu zisizopendeza zitoroke | ① | ② | ③ | ④ |
| 10 | Nimekuwa nikipata msaada na ushauri kutoka kwa watu wengine | ① | ② | ③ | ④ |
| 11 | Nimekuwa nikitumia pombe au madawa mengine kunisaidia kusahau ninayopitia | ① | ② | ③ | ④ |
| 12 | Nimekuwa nikijaribu kuangalia maisha kwa mwanga tofauti, ili kuifanya hali yangu ionekane kuwa nzuri zaidi | ① | ② | ③ | ④ |
| 13 | Nimekuwa nikijikosoa mwenyewe | ① | ② | ③ | ④ |
| 14 | Nimekuwa nikijaribu kuja na mkakati wa nini cha kufanya | ① | ② | ③ | ④ |
| 15 | Nimekuwa nikipata faraja na uelewa kutoka kwa mtu mwingine | ① | ② | ③ | ④ |
| 16 | Nimekuwa nikifanya majaribio ya kukabiliana na hali hii | ① | ② | ③ | ④ |
| 17 | Nimekuwa nikitafuta kitu kizuri katika kile kinachotokea | ① | ② | ③ | ④ |
| 18 | Nimekuwa nikifanya mzaha kuhusu hali hii | ① | ② | ③ | ④ |
| 19 | Nimekuwa nikifanya vitu vya kujisahaulisha hali hii, kama vile kwenda kwenye sinema, kutazama TV, kusoma, ndoto za mchana, kulala, au ununuzi | ① | ② | ③ | ④ |
| 20 | Nimekuwa nikikubali uhalisia wa ukweli kwamba imetokea | ① | ② | ③ | ④ |
| 21 | Nimekuwa nikielezea hisia zangu kwa hasi | ① | ② | ③ | ④ |
| 22 | Nimekuwa nikijaribu kupata faraja katika dini yangu au imani za kiroho |  | ② | ③ | ④ |
| 23 | Nimekuwa nikijaribu kupata ushauri au msaada kutoka kwa watu wengine kuhusu nini cha kufanya | ① | ② | ③ | ④ |
| 24 | Nimekuwa nikijifunza kuishi na hali hii | ① | ② | ③ | ④ |
| 25 | Nimekuwa nikifikiria sana hatua za kuchukua | ① | ② | ③ | ④ |
| 26 | Nimekuwa nikijilaumu kwa mambo yaliyotokea | ① | ② | ③ | ④ |
| 27 | Nimekuwa nikiomba au kutafakari | ① | ② | ③ | ④ |
| 28 | Nimekuwa nikifurahia hali ilivyo | ① | ② | ③ | ④ |

Ubora wa Maisha

| No. | swali | | Mbaya sana | mbaya | Sio nzuri wala mbaya | nzuri | Nzuri sana |
| --- | --- | --- | --- | --- | --- | --- | --- |
| 1 | Utapimaje ubora wa maisha yako? | | ① | ② | ③ | ④ |  |
|  |  | | Sijaridhika sana | Sijaridhika | Sijaridhika wala kutoridhika | Nimeridhika | Nimeridhika sana |
| 2 | Umeridhika vipi na afya yako? | | ① | ② | ③ | ④ | ⑤ |
| Maswali yafuatayo yanauliza kuhusu ni kiasi gani umepitia mambo fulani katika wiki mbili zilizopita | | | | | | | |
|  |  | | Sivyo hata kidogo | Kidogo | Kiasi cha wastani | Nyingi sana | Kiasi kikubwa sana |
| 3 | Kwa kiwango gani unahisi kwamba maumivu ya mwili yanakuzuia kufanya kile unachohitaji kufanya? | | ① | ② | ③ | ④ | ⑤ |
| 4 | Unahitaji kiasi gani cha matibabu ili uweze kufanya kazi katika maisha yako ya kila siku? | | ① | ② | ③ | ④ | ⑤ |
| 5 | Unafurahia maisha kwa kiasi gani? | | ① | ② | ③ | ④ | ⑤ |
| 6 | Kwa kiwango gani unahisi maisha yako kuwa na maana? | | ① | ② | ③ | ④ | ⑤ |
|  |  | | Sivyo hata kidogo | kidogo | Kidogo sana | Nyingi sana | Kiasi kikubwa sana |
| 7 | Una uwezo gani wa kua makini kuhusu suala flani? | | ① | ② | ③ | ④ | ⑤ |
| 8 | Unajisikia salama kiasi gani katika maisha yako ya kila siku? | | ① | ② | ③ | ④ | ⑤ |
| 9 | Mazingira yako ya kimwili yana afya kiasi gani? | | ① | ② | ③ | ④ | ⑤ |
| Maswali yafuatayo yanauliza kuhusu jinsi unavyopitia kabisa au uliweza kufanya mambo fulani katika wiki mbili zilizopita | | | | | | | |
|  |  | | Sivyo hata kidogo | Kidogo | Wastani | Zaidi | Kikamilifu |
| 10 | Je, una nguvu ya kutosha kwa maisha ya kila siku? | | ① | ② | ③ | ④ | ⑤ |
| 11 | Je, unaukukubali muonekano wako wa mwili? | | ① | ② | ③ | ④ | ⑤ |
| 12 | Una pesa za kutosha kukidhi mahitaji yako? | | ① | ② | ③ | ④ | ⑤ |
| 13 | Je, unaweza kufikia taarifa unazohitaji katika maisha yako ya kila siku? | | ① | ② | ③ | ④ | ⑤ |
| 14 | Kwa kiwango gani una nafasi ya kufanya shughuli za burudani? | | ① | ② | ③ | ④ | ⑤ |
|  |  | | Mbaya sana | Mbaya | Sio mbaya na si nzuri | Nzuri | Nzuri sana |
| 15 | Una uwezo wa kwenda kokote vizuri? | | ① | ② | ③ | ④ | ⑤ |
| Maswali yafuatayo yanakuuliza kusema jinsi ulivyojisikia vizuri au kuridhika kuhusu mambo mbalimbali ya maisha yako katika kipindi cha wiki mbili zilizopita | | | | | | | |
| 16 | | Umeridhika vipi na usingizi wako? | ① | ② | ③ | ④ | ⑤ |
| 17 | | Je, umeridhika vipi na uwezo wako wa kufanya shughuli zako za kila siku za maisha? | ① | ② | ③ | ④ | ⑤ |
| 18 | | Umeridhika vipi na uwezo wako wa kufanya kazi? | ① | ② | ③ | ④ | ⑤ |
| 19 | | Umeridhika vipi na wewe mwenyewe? | ① | ② | ③ | ④ | ⑤ |
| 20 | | Umeridhika vipi na mahusiano yako binafsi? | ① | ② | ③ | ④ |  |
| 21 | | Umeridhika vipi na maisha yako ya tendo la ndoa? | ① | ② | ③ | ④ | ⑤ |
| 22 | | Umeridhika vipi na msaada unaopata kutoka kwa marafiki zako? | ① | ② | ③ | ④ | ⑤ |
| 23 | | Umeridhika vipi na hali ya mahali pako pa kuishi? | ① | ② | ③ | ④ | ⑤ |
| 24 | | Je, umeridhika vipi na upatikanaji wako wa huduma za afya? | ① | ② | ③ | ④ | ⑤ |
| 25 | | Umeridhika vipi na usafiri wako? | ① | ② | ③ | ④ | ⑤ |
| Swali lifuatalo linahusu ni mara ngapi umehisi au kupitia mambo fulani katika wiki mbili zilizopita | | | | | | | |
|  | |  | sijawahi | Kwa nadra | Mara nyingi | Mara nyingi sana | Siku zote |
| 26 | | Ni mara ngapi una hisia hasi kama vile hali ya bluu, kukata tamaa, wasiwasi, unyogovu? | ① |  |  |  |  |

Mtazamo juu ya watu wenye ulemavu

| No. | Swali: Tafadhali onyesha kama unakubaliana au hukubaliani na kauli zifuatazo. | Sikubaliani nalo vikali | sikubaliani | nakubaliana | nakubaliana vikali |
| --- | --- | --- | --- | --- | --- |
| 1 | Sitaki watu wenye ulemavu wanaoishi katika mtaa wangu. | ① | ② | ③ | ④ |
| 2 | Watu wenye ulemavu wanapaswa kuchumbiana na kuoana, bila kujali kama mwenzi wake au mwenza wake ana ulemavu au la. | ① | ② | ③ | ④ |
| 3 | Watu wengi wenye ulemavu hawatabiriki na wanaonyesha tabia za msukumo. | ① | ② | ③ | ④ |
| 4 | Watu wenye ulemavu wanapaswa kuzuiwa kupata watoto. | ① | ② | ③ | ④ |

Mienendo ya Wazazi

| No. | Swali | Jibu |
| --- | --- | --- |
| 1 | Ukiondoa vitabu vya maandishi ya shule na vitabu vitakatifu, una vitabu vingapi kwa ajili ya (mtoto wako wa MWISHO au mtoto mwenye ulemavu) kusoma nyumbani? | Idadi ya vitabu   1. sina 2. vitabu 1~3 3. vitabu 4~6 4. vitabu 7~9 5. vitabu 10 au zaidi |
| 2 | Nina hamu ya kujifunza juu ya vitu ambavyo (mtoto wako wa MWISHO au mtoto mwenye ulemavu) anacheza navyo wakati akiwa nyumbani  Je, anacheza na: | |
|  | 2-1. Vinyago vya nyumbani, kama vile dolls, magari, au vinyago vingine vilivyotengenezwa nyumbani? | ① NDIYO  ② HAPANA |
|  | 2-2. Vinyago kutoka dukani au vinyago vilivyotengenezwa? | ① NDIYO  ② HAPANA |
|  | 2.3. Vitu vya nyumbani, kama vile bakuli au sufuria, au vitu vinavyopatikana nje, kama vile fimbo, miamba, magamba ya wanyama au majani? | ① NDIYO  ② HAPANA |
| 3 | Wakati mwingine watu wazima wanaowatunza watoto hulazimika kuondoka nyumbani kwenda kununua mahitaji ya nyumbani, kufua nguo, au kwa sababu nyingine na kulazimika kuacha watoto wadogo.  3-1. Kwa siku ngapi alizobaki peke yake kwa zaidi ya saa moja katika wiki iliyopita ilikuwa (mtoto wako wa mwisho au mtoto mwenye ulemavu): | 1. Hakuna 2. Siku 1 3. Siku 2~3 4. Siku 4~5 5. Siku 5~7 |
|  | 3-2. Kuachwa katika malezi ya mtoto mwingine, yaani mtu mwenye umri chini ya miaka 10, kwa zaidi ya saa moja? | 1. Hakuna 2. Siku 1 3. Siku 2~3 4. Siku 4~5 5. Siku 5~7 |

Nidhamu ya Watoto

| No. | Swali | Jibu |
| --- | --- | --- |
| 1 | 1. Watu wazima hutumia njia fulani kuwafundisha watoto tabia sahihi au kushughulikia tatizo la tabia. Nitasoma mbinu mbalimbali zinazotumika. Tafadhali niambie kama wewe au mtu mzima yeyote katika kaya yako ametumia njia hii na (mtoto wako wa MWISHO) mwezi uliopita. | |
|  | [A] Akachukua upendeleo, akakataza kitu (mtoto wako MZALIWA WA MWISHO) anavyopenda au hakumruhusu (yeye) kuondoka nyumbani. | ① NDIYO  ②HAPANA |
|  | [B] Alieleza kwa nini (tabia ya mtoto wako wa MWISHO) haikuwa sahihi. | ① NDIYO  ②HAPANA |
|  | [C] Alimtikisa | ①NDIYO  ②HAPANA |
|  | [D] Alimfokea | ① NDIYO  ②HAPANA |
|  | [E] Alimpa kitu kingine cha kufanya | ① NDIYO  ②HAPANA |
|  | [F] Alimchapa makalioni kwa mkono | ① NDIYO  ②HAPANA |
|  | [G] Alimchapa makalioni au sehemu nyingine za mwili kwa kutumia fimbo, kitana au mkanda n.k | ① NDIYO  ②HAPANA |
|  | [H] Alimuita mjinga au mvivu | ① NDIYO  ②HAPANA |
|  | [I] Alimpiga kofi usoni, na kumvuta maskio | ① NDIYO  ②HAPANA |
|  | [J] alimchapa mikononi au miguuni | ① NDIYO  ②HAPANA |
|  | [K] Alimpiga kokote kadri alivyoweza | ① NDIYO  ②HAPANA |
| 2 | **2**. Je, unaamini kwamba ili kumlea, kumlea au kumsomesha mtoto vizuri, mtoto anahitaji kuadhibiwa kimwili? | ① NDIYO  ②HAPANA |

Tathmini ya huduma za afya

Je, una mipaka gani ya kupata huduma za afya kwa mtoto wako?

| Huduma | Hakuna shida | Shida kidogo | Shida ya wastani | Shida | Tatizo lisiloweza kutatulika |
| --- | --- | --- | --- | --- | --- |
| Ukosefu wa usafiri nyumbani kwa kituo | ① | ② | ③ | ④ | ⑤ |
| Dawa au vifaa duni | ① | ② | ③ | ④ | ⑤ |
| Hakuna huduma zinazopatikana | ① | ② | ③ | ④ | ⑤ |
| Safari hatari | ① | ② | ③ | ④ | ⑤ |
| Haikuweza kumudu gharama za usafiri | ① | ② | ③ | ④ | ⑤ |
| Kiwango cha kituo cha afya | ① | ② | ③ | ④ | ⑤ |
| Upatikanaji wa wa kituo | ① | ② | ③ | ④ | ⑤ |
| Uwezo wa kumudu gharama | ① | ② | ③ | ④ | ⑤ |
| Sio mgonjwa sana | ① | ② | ③ | ④ | ⑤ |
| Mitazamo hasi wa wahudumu wa afya | ① | ② | ③ | ④ | ⑤ |
| Awali alitibiwa vibaya | ① | ② | ③ | ④ | ⑤ |
| Alijaribu lakini akakataliwa | ① | ② | ③ | ④ | ⑤ |
| Kwa sababu ya imani | ① | ② | ③ | ④ | ⑤ |
| Mawasiliano na wahudumu wa afya | ① | ② | ③ | ④ | ⑤ |
| Hakuna malazi kwenye kituo cha afya | ① | ② | ③ | ④ | ⑤ |
| tukokujua wapi pa kwenda | ① | ② | ③ | ④ | ⑤ |
| Sikuweza kupata muda wa kupumzika kazini | ① | ② | ③ | ④ | ⑤ |
| Sikua na hati muhimu | ① | ② | ③ | ④ | ⑤ |

Kufanya kazi na Ulemavu

Kama una mtoto mwenye ulemavu, mtoto wako ana shida kiasi gani kufanya shughuli hizi?

| KUNDI | | Hakuna ugumu | Ugumu fulani | Ugumu mwingi | Siwezi kufanya kabisa | Alikataa | *Sijui* |
| --- | --- | --- | --- | --- | --- | --- | --- |
| Kuona | | ① | ② | ③ | ④ | ⑦ | ⑨ |
| Kusikia | | ① | ② | ③ | ④ | ⑦ | ⑨ |
| Kutembea | | ① | ② | ③ | ④ | ⑦ | ⑨ |
| Mawasiliano katika kutumia lugha ya kawaida | | ① | ② | ③ | ④ | ⑦ | ⑨ |
| Utambuzi (kama vile kukumbuka au kutiliana maanani) | | ① | ② | ③ | ④ | ⑦ | ⑨ |
| Kujitunza (kama vile kuoga au kuvaa) | | ① | ② | ③ | ④ | ⑦ | ⑨ |
| Mwili wa juu | Kupandisha chupa ya lita 2 ya maji kutoka kiunoni hadi kiwango cha macho | ① | ② | ③ | ④ | ⑦ | ⑨ |
|  | Kutumia mikono na vidole (kama vile kuokota vitu vidogo) | ① | ② | ③ | ④ | ⑦ | ⑨ |
| Wasiwasi | Ni mara ngapi unahisi wasiwasi au mwenye mashaka | (1) Kila siku  (2) Kila wiki  (3) Kila mwezi  (4) Mara chache kwa mwaka  (5) Kamwe sijawahi | | | | ⑦ | ⑨ |
|  | Kufikiria juu ya mara ya mwisho ulipohisi wasiwasi, au mwenye mashaka ungeelezeaje kiwango cha hisia hizi? | (1) Kidogo  (2) Mengi  (3) katikati ya kidogo na mengi | | | | ⑦ | ⑨ |
| Mfadhaiko | Mara ngapi unahisi msongo wa mawazo? | (1) Kila siku  (2) Kila wiki  (3) Kila mwezi  (4) Mara chache kwa mwaka  (5) Kamwe sijawahi | | | | ⑦ | ⑨ |
|  | Kufikiria juu ya mara ya mwisho ulipohisi huzuni au msongo wa mawazo unahisi ilikua kiasi gani? | (1) Kidogo  (2) Mengi  (3) katikati ya kidogo na mengi | | | | ⑦ | ⑨ |
