# Supplementary material for: Parental stress, coping strategies, and maternal quality of life in urban Tanzania: A mixed-methods study
Source: PLoS One. 2026 Sep 25;21(9):e0358956. doi: 10.1371/journal.pone.0358956 (PMC13614557; doi:10.1371/journal.pone.0358956)
Supplement: Supplementary file 2 [file pone.0358956.s002.docx]

Inclusivity in global research

PLOS’ policy on inclusivity in global research aims to improve transparency in the reporting of research performed outside of researchers’ own country or community and ensures that PLOS publications reporting global research adhere to high standards for research ethics and authorship. Authors of relevant research articles may be asked to complete the questionnaire below, which outlines ethical, cultural, and scientific considerations specific to inclusivity in global research. This questionnaire may be requested when researchers have travelled to a different country to conduct research, if research uses samples collected in another country, research with Indigenous populations or their lands, or if research is on cultural artefacts. Researchers travelling to another country solely to use laboratory equipment will not normally be required to complete the questionnaire. However, the questionnaire can be requested at the journal’s discretion for any submission – if you have been requested to complete this questionnaire by the PLOS journal you submitted to, please do so.

Please complete the questionnaire below and include this as a Supporting Information file with your manuscript. Note that if your paper is accepted for publication, this checklist will be published with your article in the supporting information files. Please ensure that you reference the checklist in the main body of your manuscript. We suggest adding a subsection ‘Inclusivity in global research’ to your Methods section and adding the following sentence: “Additional information regarding the ethical, cultural, and scientific considerations specific to inclusivity in global research is included in the Supporting Information (SX Checklist)”

The questions have been designed to be applicable to a wide range of study types, and there are subsections for both human subjects research and non-human subjects research. If any of the questions are not relevant to your research please mark them as “N/A” as appropriate.

**Ethical considerations, permits and authorship**

*This section is applicable to all research types.*

Provide details as to who granted permissions and/or consent for the study to take place in the Methods section of your manuscript. This should include the names of **all** ethics boards, governmental organizations, community leaders or other bodies that provided approval for the study. If individuals provided approval refer to these people by their role or title but do not list their name(s).

Reported on page number: 9-10

If there were any deviations from the study protocol after approval was obtained please provide details of these changes in the Methods section of your manuscript.
Did this study involve local collaborators that are residents of the country where the research was conducted or members of the community studied? If you do not have any authors from said communities, please provide an explanation for this below.

Reported on page number: No deviations from the approved study protocol occurred after ethical approval was obtained.

Everyone listed as an author should meet PLOS’ criteria for authorship and all individuals who meet these criteria should be included in the author byline, rather than the acknowledgements. For further information please see the journal’s Authorship Policy.

**Yes.** The study was conducted in close collaboration with local administrative and health authorities in Kigamboni District, Tanzania. Prior to data collection, the research team consulted with the District Commissioner's Office and the participating health center to ensure that the study procedures were appropriate for the local context. Trained Tanzanian research assistants supported communication with local institutions, coordinated recruitment and data collection schedules, facilitated participant recruitment, provided interpretation during interviews and surveys, and helped ensure culturally appropriate implementation of the study.

Local collaborators made important contributions to participant recruitment, coordination with local institutions, scheduling, language interpretation, and culturally appropriate implementation of the study. However, they were not involved in study conception, data analysis, interpretation of findings, or manuscript drafting to the extent required to meet the PLOS authorship criteria. Accordingly, they are acknowledged in the manuscript rather than included as authors.

**Human subjects research (e.g. health research, medical research, cross-cultural psychology)**

Did you obtain written informed consent from a representative of the local community or region before the research took place? How did you establish who speaks for the community? Details of written informed consent obtained from study participants should be reported separately in the Methods section of your manuscript.

A separate informed consent form for a community representative was not used. However, prior written authorization for the study was documented through a Letter of Intent signed by the District Commissioner following a meeting with the research team. During the meeting, the research team explained the study objectives and procedures, the ethical approval obtained from the Institutional Review Board of Daegu University, and the requirement that written informed consent would be obtained from every individual participant. The District Commissioner was consulted in an official capacity as the principal administrative authority of the district, rather than as an individual speaking on behalf of every community member. Through the District Commissioner’s Office, permission to conduct the study was also obtained from the director of Kigamboni Health Centre. The District Commissioner was informed of and agreed to the proposed participant consent procedures, and written informed consent was subsequently obtained from all individual study participants before data collection.

How did members of the local community provide input on the aims of the research investigation, its methodology, and its anticipated outcome(s)?

Local administrative authorities, health professionals, and community stakeholders provided practical input during the planning and implementation of the study. Discussions with local collaborators helped ensure that the research addressed locally relevant issues related to parenting stress, coping strategies, and maternal quality of life. Local collaborators also provided practical guidance on participant recruitment, scheduling, culturally appropriate communication, and implementation of data collection.

When engaging with the local community, how did you ensure that the informed consent documents and other materials could be understood by local stakeholders?

All participant-facing materials, including informed consent forms and questionnaires, were translated into Swahili using a forward- and back-translation process. The translated materials were reviewed by bilingual experts and local health professionals to ensure linguistic accuracy, conceptual equivalence, and cultural appropriateness. During data collection, trained Tanzanian research assistants explained the study procedures in Swahili, answered participants' questions, and facilitated communication throughout the research process.

Will the findings of the research be made available in an understandable format to stakeholders in the community where the study was conducted (e.g. via a presentation, summary report, copies of publications, etc.)? Please provide details of how this will be achieved.

Study findings will be shared with participating local collaborators and institutions, including Kigamboni Health Centre, through summary reports and discussions following completion of the study. As this article will be published in an open-access journal, the findings will also be freely accessible to researchers, practitioners, policymakers, and community stakeholders in Tanzania.

**Non-human subjects research using specimens/ animals collected as part of the study, or those housed in archival collections. Examples include archaeology, paleontology, botany and zoology.**

Did the permission you obtained from a local authority to perform the study include an agreement on access to outputs and benefit sharing? This may include procedures to enable fair distribution of the benefits and resources arising from the research performed. Please include any details of Prior Informed Consent and Benefit Sharing Agreements obtained. These may be required by field-specific regulations, for example the Convention on Biological Diversity (CBD) and the associated Nagoya Protocol.

N/A (Human subjects research only).

If the material used in your study was imported, please A) provide the year it was imported and B) indicate whether permits were obtained to import/export the materials used, C) provide details of any permits obtained. If this information is not available, please indicate this.

N/A (Human subjects research only)

If you used archival specimens, please state how the material used in your study was acquired by the institute it is held in and provide details of any permits obtained for the original excavations/ sample collection. If this information is not available, please indicate this.

N/A (Human subjects research only)

How was the potential cultural significance of the materials collected in your study to local communities considered in your research design? Were Indigenous peoples and/or local researchers and institutions involved with archaeological excavations / collection of specimens? If so, please provide a description of their involvement.

N/A (Human subjects research only)

If your manuscript includes photographs of human remains please indicate whether authors obtained permission from descendants or affiliated cultural communities to do so.

N/A (Human subjects research only)
